# Citric-acid-assisted upcycling of *Sparassis latifolia* pseudosclerotia into thermoreversible polysaccharide hydrogels: Structure–property relationships and antioxidant activity *in vitro* and *in vivo*

**DOI:** 10.1016/j.fochx.2026.104447

**Published:** 2026-09-11

**Authors:** Zhiheng Qiu, Bei Jiang, Di Zhou, Jiayang Hou, Bo Bao, Yongshun An, Lu Ma, Hao Liu, Xiaoyan Zhang, Jiazhi Zhao, Xia Ren, Tianlai Li, Lili Shu

**Affiliations:** a Modern Protected Horticulture Engineering & Technology Center, College of Horticulture, Shenyang Agricultural University, Shenyang, 110866, PR China; b Key Laboratory of Protected Horticulture of Education Ministry and Liaoning Province, Shenyang, 110866, PR China; c Institute of Edible Mushroom, Fujian Academy of Agricultural Sciences & National-Local Joint Engineering Research Center for Breeding and Cultivation of Featured Edible Mushroom, Fuzhou 350011, PR China

**Keywords:** *Sparassis latifolia* pseudosclerotia, Polysaccharides, Hydrogel, Citric acid, Antioxidant activity

## Abstract

*Sparassis latifolia* pseudosclerotia represent a significant, underutilized fungal bioresource typically discarded during commercial mushroom production cycles. To upcycle this biomass, a sustainable citric-acid-assisted route transformed its extracted polysaccharides (1225 kDa; 9.74% uronic acid) into highly transparent, thermoreversible hydrogels. Rheological analysis confirmed an elastic response (*G'* > *G”*) with a macroscopic gel-to-sol transition at 80–90 °C. The hydrogel displayed an 89% water-holding capacity and 280% swelling ratio within 15 min, supported by an interconnected microstructural network. Mechanistically, physical crosslinking was driven by hydrogen bonding, hydrophobic association, and high-molecular-weight chain entanglement. The matrix exhibited measurable antioxidant-associated activity, achieving an 87.0% inhibition response in the hydroxyl-radical assay *in vitro*. *In vivo*, oral administration in mice significantly enhanced systemic redox status; high-dose intervention increased serum SOD and CAT activities by 50.1% and 114.1%, respectively, while reducing splenic malondialdehyde levels. These findings establish a basis for the further development of pseudosclerotial polysaccharides as functional food hydrocolloids.

## Highlights

• First report of a citric acid-induced hydrogel from *S. latifolia* pseudosclerotia polysaccharide.
• The resulting transparent hydrogel exhibits excellent thermoreversibility.
• The gelation is driven by physical cross-llinks rather than covalent bonds.
• The hydrogel exhibited strong antioxidant activities both *in vitro* and *in vivo*.

## 1 Introduction

*Sparassis latifolia*, commonly known as the cauliflower mushroom, is a rare yet widely cultivated edible and medicinal mushroom in East Asia. It has attracted growing attention as a promising source of bioactive macromolecules for food and wellness products. Polysaccharides, among its major constituents, are particularly abundant and have demonstrated a broad spectrum of biological activities in the *Sparassis* genus, including antioxidant, anti-inflammatory, immunomodulatory, and anti-tumor effects (Qiu et al., 2025). Specifically, its polysaccharides are predominantly *β*-glucans with (1 → 3)- and (1 → 6)-linkages (Hida et al., 2013). Such polysaccharides are of particular interest in food hydrocolloid science due to their water-binding capacity, thickening properties, and potential to form or modify gel networks. Despite this promising composition, studies on the gelation behavior within the genus remain limited. To date, research on gel-forming polysaccharides has focused largely on *S. crispa* (Furuta et al., 2023), while systematic evidence from *S. latifolia* is still scarce. This knowledge gap not only diminishes the recognition of *S. latifolia* as a viable hydrocolloid source but also limits its potential application in food structuring technologies. Moreover, to meet the growing demand for multifunctional polysaccharides in the food industry, it is important to identify and investigate new polysaccharides with potential applications.

Commercial production of *S. latifolia* has expanded considerably in China, with an annual fruiting-body production of approximately 6971 t reported in 2023. The cultivation process, however, simultaneously generates a substantial amount of pseudosclerotial biomass (Hughes et al., 2013). Based on production records from the commercial cultivation facility supplying the material used in this study, each cultivation bag typically produces approximately 150–200 g of fresh fruiting bodies, accompanied by approximately 50–67 g of pseudosclerotia. The fresh weight of the pseudosclerotia therefore corresponds to approximately one-third of the fruiting-body yield. Extrapolation of this production ratio to the reported national fruiting-body output suggests that approximately 2.3 × 10^3^ t of fresh pseudosclerotial biomass may be generated annually, although this value should be regarded as a production-based estimate rather than an official national statistic. At present, this compact mycelial biomass is largely underutilized after mushroom harvesting, resulting in the loss of potentially valuable fungal resources. From a circular-bioeconomy perspective, *S. latifolia* pseudosclerotia may provide an alternative source of polysaccharides for the development of value-added food hydrocolloids. To our knowledge, their conversion into functional polysaccharide hydrogels has not yet been systematically investigated.

The gel-forming ability of polysaccharides is a highly desirable property that finds broad applications (Lebedieva et al., 2025). This functionality is directly utilized in food processing as gelling agents and thickeners, in pharmaceuticals for drug delivery systems, and in biomaterial science for tissue engineering hydrogels (Hakimi et al., 2025; Wang et al., 2025, Wang et al., 2025). The gelation of food hydrocolloids is frequently orchestrated by physicochemical cues that balance polymer–solvent and polymer–polymer interactions (Li, Fang, et al., 2023). A key method is acid-induced gelation, which leverages simple pH adjustment through food acids or controlled fermentation to form gels (Lin et al., 2024). This clean-label approach offers a notable advantage by proceeding under mild conditions without requiring exogenous crosslinkers. Recent research on natural polysaccharide-based hydrogels has shown that acid-induced gelation is a viable and green strategy to create functional materials, as exemplified by the development of a pH-responsive hydrogel from *Poria cocos* polysaccharide with excellent biocompatibility and antioxidant activity (Li, Liu, et al., 2023). Strong electrostatic hydrogels were successfully prepared by slow acidification of amaranth protein and xanthan gum mixtures with glucono-δ-lactone, demonstrating that maximal associative electrostatic interactions, which are crucial for gel network formation, occur at a pH below the protein's isoelectric point (Cortez-Trejo et al., 2022). While polysaccharide-based hydrogels are attractive for biomedical uses, their mechanical shortcomings persist. Innovative crosslinking strategies are being developed to address this, such as the use of CA, which acts as both an extractant and a cross-linker for ulvan polysaccharide from *Ulva sp.* macroalgae. This esterification-based process not only achieves a high extraction yield but also produces hydrogels with excellent swelling capacity (Manikandan & Lens, 2022). Despite these advancements in polysaccharide gelation, the discovery and characterization of novel gelling polysaccharides from sustainable and underutilized biomass, particularly those capable of forming gels through simple and green processes like acidification, remain an attractive and ongoing pursuit in the field.

We hypothesized that the high molecular weight and uronic-acid-containing characteristics of SLPPs would favor the formation of a thermoreversible physical network under a defined citric-acid-assisted thermal treatment. Specifically, we investigated whether citric-acid-assisted treatment, followed by heating and cooling, could promote reversible intermolecular association and chain entanglement, thereby generating a stable three-dimensional hydrogel with favorable water-management and mechanical properties. The physicochemical characteristics, intermolecular interactions, thermoreversible behavior, and antioxidant-related properties of the resulting hydrogel were subsequently evaluated. These findings provide a feasible strategy for the sustainable valorization of mushroom cultivation by-products while introducing a new, natural gelling agent with significant potential for applications in the food, cosmetic, and pharmaceutical industries. These findings may support the development of functional products based on *S. latifolia* and enhance the overall value chain of its production.

## 2 Materials and methods

### 2.1 Materials and reagents

The SLP was obtained from Fujian Rongyi Mushroom Technology R&D Co., Ltd. (Fujian, China). The SLP samples were freeze-dried (Christ ALPHA 1-2LD plus, Martin Christ Gefriertrocknungsanlagen GmbH, Osterode am Harz, Germany), and then ground into a fine powder. Subsequently, the powder was passed through a 60-mesh sieve. Citric acid (anhydrous), galacturonic acid, dextran, D-(+)-glucose, Sepharose CL-4B, and Bradford protein assay kit were purchased from Sigma Chemical Co. (St. Louis, Mo, USA). Macroporous resins (D-900) were purchased from RuiDaHengHui Technology Co., Ltd. (Beijing, China). 2,2-diphenyl-1-picrylhydrazyl (DPPH), 2,2′-azino-bis (3-ethylbenzothiazoline-6-sulfonic acid) diammonium salt (ABTS), and l-ascorbic acid were purchased from Aladdin Biochemical Technology Co., Ltd. (Shanghai, China). All other reagents and chemicals were of analytical grade and were obtained from Sangon Biotech (Shanghai) Co., Ltd. (Shanghai, China).

### 2.2 Extraction of SLPPs

**Fig. 1:**
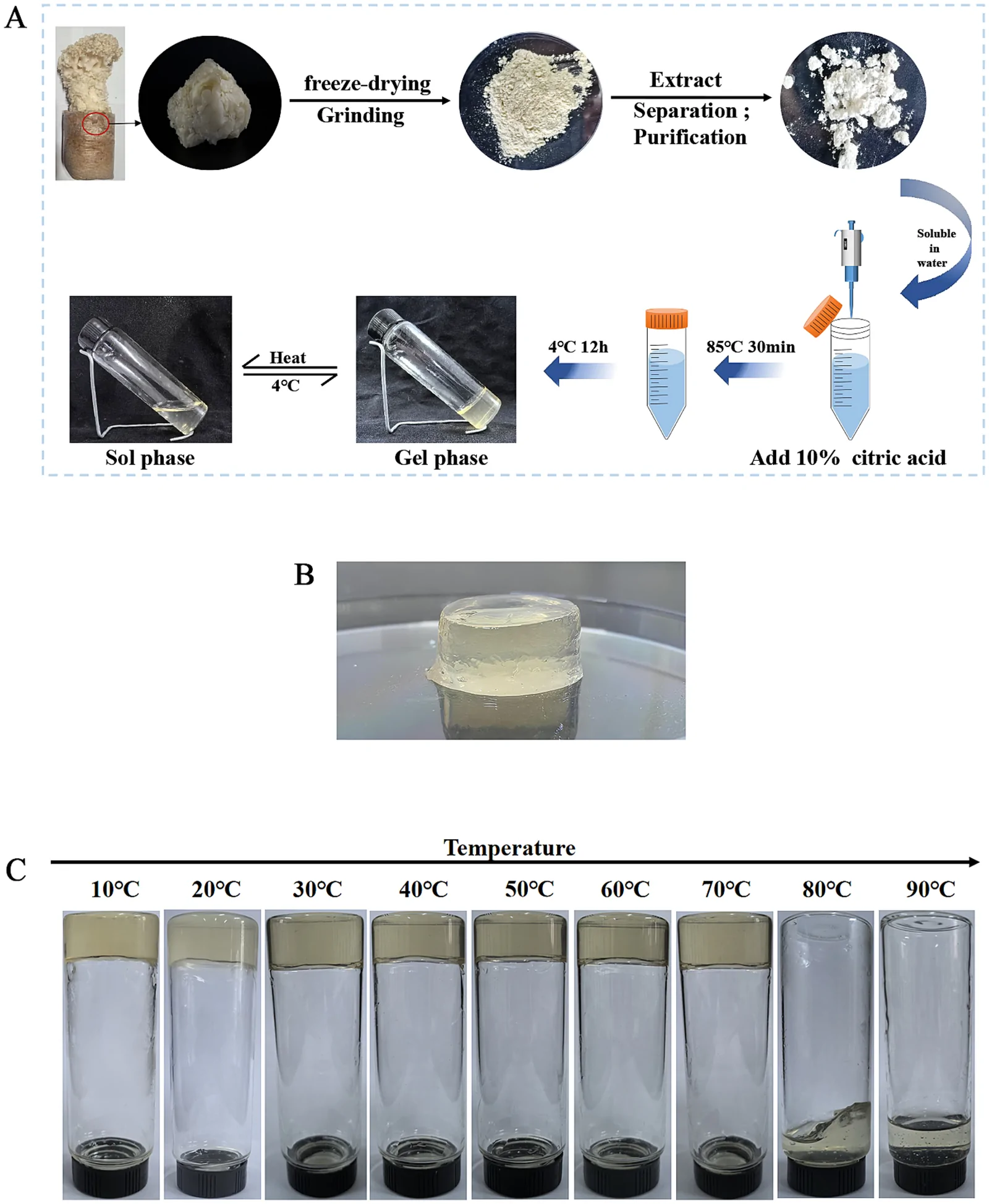
Schematic illustration of the extraction and purification of SLPPs, followed by citric acid-induced hydrogel formation and its characteristic thermoreversible behavior. (A) Process flow from raw material to sol and final gel. (B) Macroscopic appearance of the obtained transparent hydrogel. (C) The thermoreversible behavior of the SLPPs hydrogel across a temperature gradient (10–90 °C).

The extraction of SLPPs was carried out according to a previous method with modifications (Fig. 1A) (Fei et al., 2024). Briefly, the powder (10 g) was mixed with distilled water at a solid-to-liquid ratio of 1:50. The mixture was then extracted twice in a water bath at 90 °C for 4 h, with stirring at 1 h intervals. After extraction, the mixture was centrifuged at 12,000 ×*g*, and the supernatant was collected. Proteins were removed from the supernatant using the Sevag reagent. The solution was then concentrated and mixed with four volumes of ethanol to precipitate the polysaccharides at 4 °C overnight. The resulting precipitate was collected by centrifugation at 12,000 ×*g*, yielding the crude polysaccharide. Finally, the crude polysaccharide was freeze-dried and ground into a powder.

### 2.3 Isolation and purification of SLPPs

The decolorization of crude SLPPs was carried out on a D-900 macroporous resin column. A 10 mL sample of the crude SLPPs solution (200 mg/mL) was applied to the column. After elution with ultrapure water, the resulting eluate was collected. The eluate was transferred to a vacuum freeze dryer to obtain the lyophilized polysaccharide powder. Briefly, the freeze-dried crude polysaccharides were dissolved in ultrapure water to prepare a 2 mg/mL solution. The solution was filtered through a 0.45 μm microporous membrane. The filtrate was then loaded onto a DEAE-52 cellulose column at a flow rate of 1 mL/min. Stepwise elution was performed using NaCl solutions at increasing concentrations (0, 0.1, 0.3, and 0.5 M). The eluates were collected fractionally based on the elution profile. The polysaccharide content in the collected fractions was determined by the phenol‑sulfuric acid method to plot the elution curve (Wen-Hui et al., 2019). The major fractions corresponding to each peak were pooled separately, concentrated, dialyzed (*Mw* cut-off: 3500 Da) against ultrapure water to remove salts, and finally lyophilized. The fraction with the highest yield was selected for all subsequent experiments and analyses.

The fraction was further purified using Sepharose CL-4B gel filtration chromatography resin. The column was pre-equilibrated with 0.1 M NaCl solution. After loading the sample, elution was performed using the same 0.1 M NaCl solution at a constant flow rate of 0.5 mL/min. The elution profile was monitored, and fractions corresponding to the major peak were collected, pooled, dialyzed (*Mw* cut-off of 3500 Da) and lyophilized to obtain the purified sample.

### 2.4 UV spectral analysis of SLPPs

SLPPs (10 mg) were dissolved in distilled water to prepare a 1.0 mg/mL aqueous solution. The UV absorption spectrum of the SLPPs solution was recorded over the wavelength range of 200–500 nm using a U-5100 UV–Vis spectrophotometer (HITACHI, Japan), with distilled water used as the blank.

### 2.5 Molecular weight analysis

The molecular weight of SLPPs was determined by gel permeation chromatography (GPC) using a system equipped with a Waters 1515 pump and a 2707 autosampler. Separation was achieved using SB-806 M and SB-804 columns connected in series, with 0.1 M sodium nitrate serving as the mobile phase at a flow rate of 0.6 mL/min and a column temperature of 40 °C. The detection system consisted of a Wyatt Technologies DAWN Heleos multi-angle light scattering (MALS) detector (operating at a wavelength of 658 nm), a ViscoStar differential viscometer, and an Optilab T-rEX differential refractometer. The SLPPs sample was prepared by dissolving it in the mobile phase at a concentration of 1 mg/mL, followed by filtration through a 0.45 μm membrane filter before injection. Data were collected and processed using Astra software (version 6.1.4, Wyatt Technologies), with a specific refractive index increment (dn/dc) value of 0.138 mL/g applied for the analysis.

### 2.6 Chemical composition analysis of SLPPs

#### 2.6.1 Total sugar content

The total sugar content in SLPPs was determined by the phenol‑sulfuric acid method (Zhao et al., 2025).

#### 2.6.2 Uronic acid content

The uronic acid content in SLPPs was determined using the sulfuric acid-carbazole colorimetric method (Zhang et al., 2023). Galacturonic acid was used as the standard.

#### 2.6.3 Protein content

The protein content of SLPPs was determined using a Bradford method protein assay kit.

#### 2.6.4 Monosaccharide composition analysis

The monosaccharide composition of SLPPs was analyzed by PMP pre-column derivatization followed by HPLC (Liu et al., 2021). Standard monosaccharides, including rhamnose (Rha), arabinose (Ara), galactose (Gal), glucose (Glc), xylose (Xyl), mannose (Man), galacturonic acid (GalA), glucuronic acid (GlcA), glucosamine hydrochloride (GlcN), and galactosamine hydrochloride (GalN), were prepared individually.

### 2.7 Preparation of SLPPs hydrogel under the selected citric-acid-assisted condition

A reproducible formulation condition was selected to investigate the gel-forming behavior and physicochemical properties of SLPPs. Lyophilized SLPPs were dispersed at 2.0 wt% in 10% (*w*/*v*) citric acid solution, heated at 85 °C for 30 min, and subsequently maintained at 4 °C for 12 h to obtain a self-supporting hydrogel. For pH determination, 1.0 g of freshly prepared hydrogel was dispersed in 10 mL of deionized water and equilibrated for 30 min before measurement using a calibrated pH meter. The apparent pH of the hydrogel dispersion was 2.76 ± 0.09 (*n* = 3).

Before antioxidant evaluation, the formed hydrogels were thoroughly washed with deionized water to remove free and readily diffusible citric acid. Briefly, 1 g of hydrogel was washed with 10 mL of deionized water for 10 min and the washing procedure was repeated 5 times. Following washing, 1.0 g of hydrogel was dispersed in 10 mL of deionized water, equilibrated for 30 min, and its apparent pH was determined. The washed hydrogel dispersion exhibited an apparent pH of 5.26 ± 0.06. The washed hydrogel was subsequently used for the antioxidant assays.

### 2.8 Thermoreversible behavior of SLPPs hydrogel

To evaluate the thermal reversibility, the prepared SLPPs hydrogel was subjected to sequential incubation in water baths at temperatures ranging from 10 to 90 °C (at 10 °C intervals) for 10 min at each temperature. Subsequently, the samples were cooled at 4 °C for 30 min. The state transitions of the gel were visually observed throughout the entire heating and cooling cycle.

### 2.9 Determination of water-holding characteristics of SLPPs hydrogel

The water-holding capacity (WHC) of SLPPs hydrogel was determined by centrifugation according to a previous method (Zhao et al., 2025). Briefly, approximately 2 g of the sample was wrapped in double-layer filter paper, placed in a 50 mL centrifuge tube, and centrifuged at 10,000 rpm for 15 min at 4 °C. After centrifugation, the surface water was removed with the filter paper. The WHC was then calculated using Eq. (1).

(1) $$\mathrm{WHC}\;\left(\%\right)=\frac{M_{0}}{M_{1}}\times 100\%\#$$

*M*_0_ - mass of the hydrogel sample after centrifugation (g).

*M*_1_ - mass of the hydrogel sample before centrifugation (g).

Measurements were performed using three independently prepared hydrogel batches (*n* = 3).

### 2.10 Swelling behavior of SLPPs hydrogel

The swelling behavior of SLPPs hydrogel was evaluated by a gravimetric method (Zhang et al., 2021). Briefly, 1 mL of each hydrogel was prepared in a 24-well plate and lyophilized at −55 °C. The resulting lyophilized gels were weighed (*W*_0_) and placed on a 200-mesh sieve. The samples were then immersed in phosphate-buffered saline (PBS; 0.02 M, pH 7.4) at 25 °C. At predetermined time points, the hydrogels were removed, gently blotted dry to remove surface moisture, and weighed to record the swollen mass (*W*_t_). The swelling ratio (SR) was calculated using Eq. (2).

(2) $$\begin{array}{c} \mathrm{SR}\;\left(\%\right)=\frac{\mathrm{Wt}-W0}{W0}\times 100\%\# \end{array}$$

*W*_t_: the weight after swelling, measured at predetermined intervals.

*W*_0_: the initial weight before swelling.

Measurements were performed using three independently prepared hydrogel batches (*n* = 3).

### 2.11 Low-field nuclear magnetic resonance (LF-NMR) of SLPPs hydrogel

The water state in the SLPPs hydrogel system was analyzed using LF-NMR with a MacroMR12-150H-I instrument (Shanghai, China) according to a previous method (Zhao et al., 2025). The sample was placed on a 60 mm track and inserted into the coil for measurement of the spin-spin relaxation time (*T*_2_). The instrument was standardized and calibrated prior to analysis. The typical pulse parameters were as follows: a 90° pulse width of 13 μs, a 180° pulse width of 26.48 μs, a frequency offset of 54,014.51 Hz, a main frequency of 21 MHz, a sampling frequency of 200 kHz, an RF delay of 0.002 ms, an analog gain of 20, a digital gain of 3, a preamplification factor of 2, an echo time of 0.2 ms, 10,000 echoes, and 8 accumulations. The CPMG decay curves were fitted using Multi Exp Lnv analysis software with multi-exponential fitting and SRIT algorithms, with the number of inversion iterations set to 100,000.

### 2.12 Fourier transform infrared (FT-IR)

SLPPs hydrogel was thoroughly mixed with dry KBr at a mass ratio of 1:30 (*w*/w), finely ground, and then compressed into pellets. FT-IR spectra of the SLPPs hydrogel were obtained using an iS50 Nicolet FTIR spectrometer with 64 scans over the range 4000–400 cm^−1^ (Tian et al., 2024).

### 2.13 Texture analysis of SLPPs hydrogel

#### 2.13.1 Texture profile analysis (TPA)

The gel sample was equilibrated on the texture analyzer platform at room temperature for 10 min. Texture profile analysis was then performed using a TMS-Pilot instrument equipped with a P/36R cylindrical probe. The test parameters were set as follows: trigger force, 0.75 N; target strain, 50%; pre-test and post-test speeds, 60 mm/min; and lift-off height, 15 mm.

#### 2.13.2 Gel strength

The gel strength was determined by a puncture test with a 0.5 mm probe after a 10 min equilibration at room temperature. The test was conducted with the following settings: a trigger force of 0.75 N, a puncture distance of 10 mm, a test speed of 60 mm/min, and a lift-off height of 15 mm.

#### 2.13.3 Rheological properties of SLPPs hydrogel

The rheological properties of the SLPPs hydrogel system were characterized using an MCR 302e rheometer (Anton Paar, Austria) according to a previous method (Xia et al., 2018). First, amplitude sweeps (0.1–100% strain) at 1 Hz were carried out to determine the linear viscoelastic region (LVR) by monitoring the energy storage modulus (*G*′) and loss modulus (*G*″). Then, within the determined LVR, frequency sweeps were performed over a frequency range of 0.1 to 100 rad·s^−1^ to evaluate the gel's viscoelastic behavior as a function of angular frequency.

### 2.14 Gelling interactions

The molecular interactions governing the formation of SLPPs hydrogel were investigated according to a previously reported method with modifications (Li, Liu, et al., 2023). To probe the contribution of specific interactions to gelation, different disrupting agents—SDS (for hydrophobic interactions), urea (for hydrogen bonding), and NaCl (for electrostatic interactions)—were introduced during hydrogel preparation. The final concentrations of SDS and urea were 0.01, 0.02, 0.03, 0.04 and 0.05 wt%, respectively, while NaCl was tested at 0.1, 0.2, 0.3, 0.4, and 0.5 M. The sol–gel transition behavior of SLPPs hydrogels was then systematically evaluated under each condition to assess the gel formation capacity.

### 2.15 Thermal stability analysis of SLPPs hydrogel

The thermal stability of the SLPPs hydrogel system was evaluated by thermogravimetric analysis (TGA) (Zhao et al., 2025). TGA was performed on a TGA 8000™ thermogravimetric analyzer (Perkin Elmer, USA). Approximately 5 mg of the lyophilized hydrogel powder was placed in an alumina crucible and heated from 50 to 600 °C at a rate of 10 °C/min under a nitrogen atmosphere with a flow rate of 20 mL/min.

### 2.16 Scanning electron microscopy (SEM)

The microstructures of SLPPs and the SLPPs hydrogel were examined using a Hitachi S-4800 scanning electron microscope (Japan). Lyophilized samples were mounted on a cylindrical stub with conductive adhesive and sputter-coated with a thin layer of gold under vacuum. Micrographs were acquired at 200×, 500×, and 1000× magnification to visualize the internal morphology of the gel network.

### 2.17 Antioxidant activities of the SLPPs hydrogel *in vitro*

The antioxidant activities of the SLPPs hydrogel were evaluated by measuring the scavenging capabilities against DPPH, ABTS, and hydroxyl radicals, following previously reported methods with slight modifications (Aguanell et al., 2022; Dziadek et al., 2022). Unless otherwise specified, the hydrogel used in all antioxidant assays was the washed hydrogel obtained after the post-gelation washing procedure described above. Three independently prepared hydrogel batches were used for each assay (*n* = 3), with technical measurements performed in triplicate where applicable. A 20 μg·mL^−1^ l-ascorbic acid (VC) solution was included as a positive assay reference. Each tested hydrogel sample was derived from a formulation containing 20 mg of SLPPs per 1.0 mL hydrogel before post-gelation washing. The apparent pH of the washed hydrogel dispersion was 5.26 ± 0.06.

#### 2.17.1 DPPH radical scavenging assay

Three milliliters (3 mL) of 100 μM DPPH in ethanol was added to each hydrogel sample and incubated at 30 °C for 30 min in the dark. The absorbance of the solution was measured at 515 nm using a UV–Vis spectrophotometer (U-5100, HITACHI, Japan). The DPPH radical scavenging activity was calculated as follows (Eq. 3):

(3) $$\begin{array}{c} \text{DPPH scavenging}\;\left(\%\right)=\frac{A_{\mathrm{blank}}-A_{\mathrm{sample}}}{A_{\mathrm{blank}}}\times 100\%\# \end{array}$$

where *A*_*blank*_ and *A*_*sample*_ represent the absorbance of the control (DPPH solution with ethanol) and the test sample (DPPH solution with ethanol and SLPPs hydrogel), respectively.

#### 2.17.2 ABTS radical scavenging assay

The ABTS radical cation solution was prepared by mixing equal volumes of 7 mM ABTS aqueous solution and 2.45 mM aqueous potassium persulfate solution, followed by incubation in the dark at 25 °C for 12 h. Subsequently, 3 mL of the ABTS solution was added to the hydrogel and kept at 30 °C for 30 min in the dark. The absorbance was measured at 734 nm. The ABTS radical scavenging activity was calculated as follows (Eq. 4):

(4) $$\begin{array}{c} \text{ABTS scavenging}\;\left(\%\right)=\frac{A_{\mathrm{blank}}-A_{\mathrm{sample}}}{A_{\mathrm{blank}}}\times 100\%\# \end{array}$$

where *A*_*blank*_ and *A*_*sample*_ denote the absorbance of the ABTS radical cation solution without and with the hydrogel, respectively.

#### 2.17.3 Hydroxyl radical scavenging assay

To evaluate hydroxyl radical scavenging activity, 1 mL of 2.5 mM salicylic acid (in ethanol), 1 mL of 5 mM ferrous sulfate (aqueous), and 1 mL of 5 mM hydrogen peroxide (aqueous) were successively added to the hydrogel and incubated at 30 °C for 30 min. Absorbance was measured at 536 nm. The hydroxyl radical scavenging activity was calculated as follows (Eq. 5):

(5) $$\begin{array}{c} \text{Hydroxyl scavenging}\;\left(\%\right)=\frac{A_{\mathrm{blank}}-A_{\mathrm{sample}}}{A_{\mathrm{blank}}}\times 100\%\# \end{array}$$

where *A*_*blank*_ and *A*_*sample*_ refer to the absorbance of the control (salicylic acid + ferrous sulfate + H_2_O_2_) and the test sample (hydrogel + salicylic acid + ferrous sulfate + H_2_O_2_), respectively.

To evaluate whether the mildly acidic pH of the washed hydrogel affected the spectrophotometric antioxidant assays, preliminary control experiments were conducted over the pH range of 5.0–6.0. The corresponding DPPH and ABTS control systems were adjusted within this pH range under otherwise identical assay conditions, and their absorbance values were compared (Table S1). No significant differences were detected among the pH-adjusted controls (*P* > 0.05), indicating that variation within this pH interval had negligible influence on the baseline absorbance of the DPPH and ABTS assay systems.

### 2.18 Antioxidant activities of the SLPPs hydrogel *in vivo*

All animal procedures were conducted in accordance with the Chinese national guideline GB/T 35892–2018 (Laboratory animal—Guideline for ethical review of animal welfare). The study design also referred to internationally recognized principles of Directive 2010/63/EU, the NIH Guide (NIH Publication No. 85–23, revised 1996), and the ARRIVE guidelines. Every effort was made to minimize pain and distress and to use the minimum number of animals necessary to achieve scientific validity. Approval for the study protocol was obtained from Ethical Review Committee and Laboratory Animal Welfare Committee of Shenyang Agricultural University (Shenyang, China) (Approval number: SNLL26022701).

For *in vivo* treatment, the polysaccharide hydrogel was homogenized into a uniform dispersion in sterile 0.8% saline using a high-shear homogenizer (10,000 rpm, 60 s) to ensure consistent dosing. Male C57BL/6 J mice (6–8 weeks old, 18–22 g) were obtained from an accredited vendor and acclimatized for 7 days before the experiment. Mice were housed under standard conditions (22 ± 2 °C, 50–60% humidity, 12 h light/dark cycle) with *ad libitum* access to chow and water. They were randomly assigned to five groups: a normal control (NC) group receiving 0.8% (*w*/*v*) saline (0.3 mL/day), a positive control group receiving VC, and three hydrogel-treated groups receiving low (HL), medium (HM), and high doses (HH) of the hydrogel (30, 120, and 300 mg·kg^−1^ body weight, respectively; 0.3 mL/day) (He et al., 2023). Each group contained twelve mice, and treatments were administered by oral gavage once daily for 30 days. Throughout the 30-day intervention, the animals were routinely observed for mortality and overt abnormalities in general condition or behavior.

At the end of the intervention, mice were fasted for 24 h with free access to water. Blood was collected by retro-orbital sampling. After centrifugation at 4 °C and 5000 rpm for 8 min, the supernatant was collected for subsequent analysis. Animals were then euthanized, and the liver and spleen were excised, rinsed with ice-cold saline, blotted dry, and weighed.

Serum was analyzed directly for superoxide dismutase (SOD) activity, catalase (CAT) activity, and malondialdehyde (MDA) content. For tissue analysis, 10% (*w*/*v*) liver and spleen homogenates were prepared in physiological saline, and the corresponding homogenate supernatants were used for biochemical measurements. The total protein concentration of each tissue-homogenate supernatant was determined using a BCA protein assay kit (Suzhou Comin Biotechnology Co., Ltd., Suzhou, China) and expressed as mg·mL^−1^. SOD and CAT activities and MDA contents in tissue samples were normalized to the total protein concentration of the corresponding homogenate supernatant. All absorbance measurements were performed using a UV–Vis spectrophotometer. SOD, CAT, MDA, and BCA protein assay kits were obtained from Suzhou Comin Biotechnology Co., Ltd. (Suzhou, China) and used according to the manufacturer's instructions.

For SOD determination, the inhibition fraction (*I*) was calculated as:

(6) $$\begin{array}{c} I=\frac{\;A_{\mathrm{control}}-A_{\mathrm{sample}}}{A_{\mathrm{control}}}\# \end{array}$$

Serum SOD activity was calculated as:

(7) $$\begin{array}{c} \mathrm{SOD}\;\text{activity}\;\left(U\cdot {\mathrm{mL}}^{-1}\right)=\frac{\;11.4I}{1-I}\# \end{array}$$

and tissue SOD activity was calculated as:

(8) $$\begin{array}{c} \mathrm{SOD}\;\text{activity}\;\left(U\cdot {\mathrm{mg}}^{-1}\text{protein}\right)=\frac{\;11.4I}{\left(1-I\right)C_{\mathrm{pr}}}\# \end{array}$$

where *C*_*pr*_ is the protein concentration of the tissue-homogenate supernatant (mg·mL^−1^).

For CAT determination, the absorbance difference was calculated as:

(9) $$\begin{array}{c} ⊿A=A_{\mathrm{control}}-A_{\mathrm{sample}}\# \end{array}$$

Serum CAT activity was calculated as:

(10) $$\begin{array}{c} \mathrm{CAT}\;\text{activity}\;\left(U\cdot {\mathrm{mL}}^{-1}\right)=8.9\;\left(⊿A-0.0013\right)\# \end{array}$$

and tissue CAT activity was calculated as:

(11) $$\begin{array}{c} \mathrm{CAT}\;\text{activity}\;\left(U\cdot {\mathrm{mg}}^{-1}\text{protein}\right)=\frac{8.9\;\left(⊿A-0.0013\right)}{C_{\mathrm{pr}}}\# \end{array}$$

For MDA determination:

(12) $$\begin{array}{c} ⊿A=A_{532}-A_{600}\# \end{array}$$

Serum MDA content was calculated as:

(13) $$\begin{array}{c} \mathrm{MDA}\;\left(\text{nmol}\cdot {\mathrm{mL}}^{-1}\right)=25.8⊿A\# \end{array}$$

and tissue MDA content was calculated as:

(14) $$\begin{array}{c} \mathrm{MDA}\;\left(\text{nmol}\cdot {\mathrm{mg}}^{-1}\text{protein}\right)=\frac{25.8⊿A}{C_{\mathrm{pr}}}\# \end{array}$$

### 2.19 Statistical analysis

Data are expressed as mean ± standard deviation (SD). Unless otherwise stated, quantitative physicochemical and *in vitro* functional measurements were performed using three independently prepared hydrogel batches (*n* = 3). Each hydrogel batch was prepared independently following the same formulation procedure and constituted one experimental replicate. Where multiple analytical or instrumental measurements were obtained from the same batch, these were treated as technical replicates and averaged before statistical analysis. For the animal experiment, each mouse was considered an independent biological replicate (*n* = 12 per treatment group). Repeated analytical measurements obtained from the same biological sample were averaged before inferential analysis.

Normality was evaluated using the Shapiro–Wilk test, and homogeneity of variance was assessed using Levene's test. Data satisfying normality and homoscedasticity were analyzed by one-way ANOVA followed by Tukey's honestly significant difference (HSD) test. When homogeneity of variance was not satisfied, Welch's ANOVA followed by the Games–Howell test was applied. Data not satisfying parametric assumptions were analyzed using the Kruskal–Wallis test followed by Dunn's multiple-comparison test. Statistical significance was set at *P* < 0.05. Statistical analyses were performed using SPSS 26.0.

## 3 Results and discussion

### 3.1 Isolation and purification of SLPPs

**Fig. 2:**
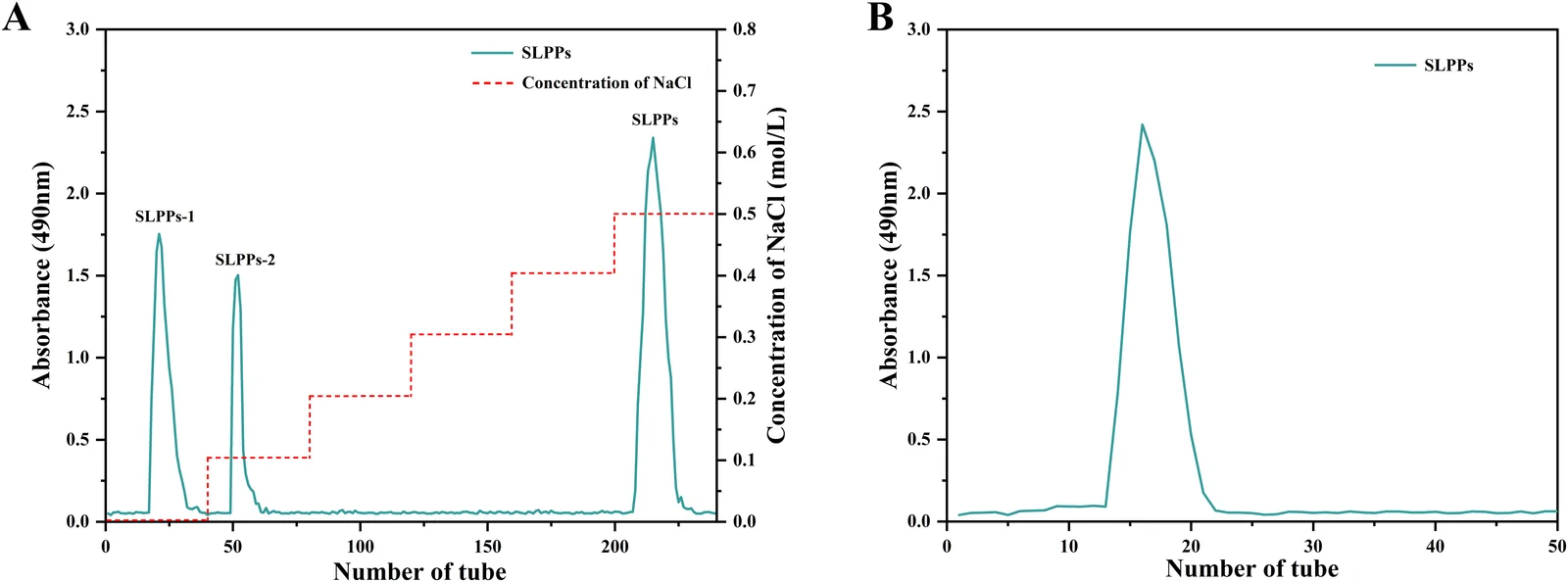
Isolation and purification elution cures of SLPPs. (A) DEAE-52 cellulose gradient elution curves. (B) Sepharose CL-4B elution curve.

As shown in Fig. 2A, the crude polysaccharide fraction was separated into three components, designated as SLPPs, SLPPs-1, and SLPPs-2, on a DEAE-52 ion exchange column. SLPPs-1 exhibited an elution profile with ultrapure water, whereas SLPPs-2 and SLPPs displayed gradient elution profiles with 0.1 M and 0.5 M NaCl solutions, respectively. Consequently, the SLPPs fraction with the highest yield (45.30%) was collected for subsequent purification. As shown in Fig. 2B, SLPPs exhibited a homogeneous elution profile on Sepharose CL-4B dextran gel. The corresponding single peak was collected, concentrated, and lyophilized to obtain the purified SLPPs.

### 3.2 Chemical composition and *mw* of SLPPs

**Supplementary Fig. S1:**
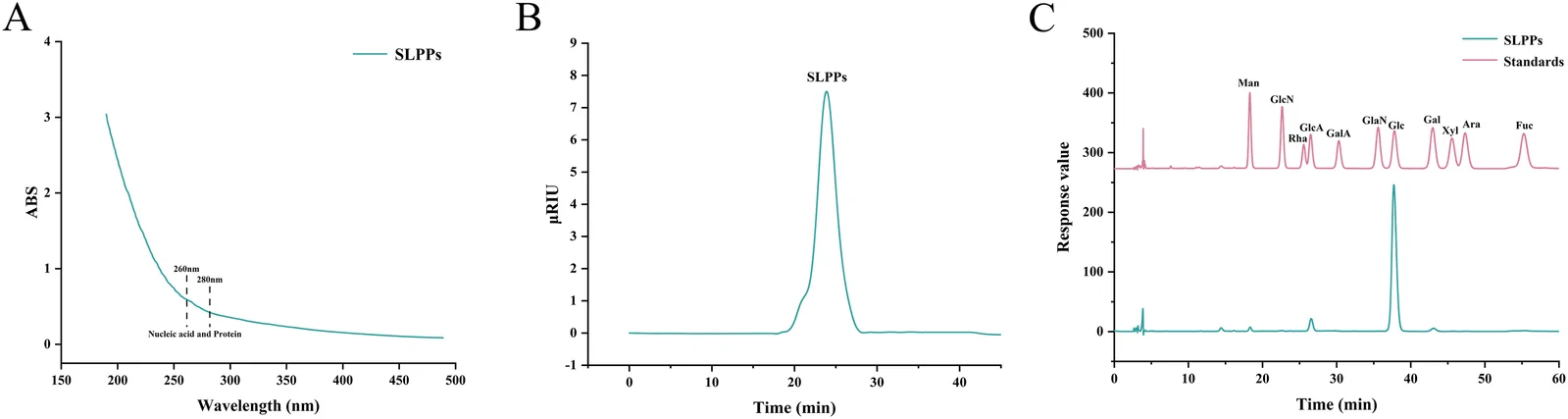
Purity and structural characterization of SLPPs. (A) UV absorption spectrum of SLPPs. (B) HPGPC profile of SLPPs. (C) Monosaccharide composition of SLPPs.

The chemical composition and molecular characteristics of the extracted SLPPs are summarized in Table 1. The total sugar content of SLPPs was 78.93%, indicating that the extraction and purification procedure efficiently recovered carbohydrate-rich components from SLP. To further evaluate the purity of SLPPs, the UV absorption spectrum of the purified sample was recorded over the range of 200–500 nm. As shown in Fig. S1A, no obvious absorption peaks were observed at 260 or 280 nm, indicating the absence of detectable nucleic acid and protein residues in the purified polysaccharide fraction. This result suggests that the extraction and purification procedures effectively removed residual biomolecules, further supporting the relatively high purity of SLPPs and providing a reliable basis for subsequent structural and functional analyses. The uronic acid content was 9.74%, suggesting that SLPPs contained a considerable proportion of acidic sugar residues, which may be closely associated with their gelation behavior under acidic conditions. In contrast, the protein content was only 0.35%, indicating effective deproteinization and minimizing the potential interference of proteins in the physicochemical behavior of the polysaccharide system. The molecular characteristics of SLPPs were further analyzed by HPGPC. As shown in Fig. S1B, SLPPs exhibited a major chromatographic peak at a retention time of 23.8 min, suggesting that the purified sample possessed a relatively concentrated molecular size distribution. Based on the calibration curve established using polysaccharide standards with different molecular weights, the molecular weight of SLPPs was calculated to be 1225 kDa. The dispersity (Đ = *Mw*/*Mn*) was 1.43, suggesting a relatively narrow molecular weight distribution and reasonably uniform chain-length distribution of the extracted polysaccharide (Wang et al., 2018). This result indicates that SLPPs is an ultra-high-molecular-weight polysaccharide, which may favor intermolecular entanglement and association and thus contribute to the formation of a stable three-dimensional hydrogel network under acidic conditions (Wang et al., 2025b).

**Table 1:** Chemical composition and *Mw* of SLPPs.

Sample	Total sugar (%)	Uronic acid (%)	Protein (%)	*Mw* (kDa)	*Mw*/*Mn*
SLPPs	78.93 ± 0.72	9.74 ± 0.33	0.35 ± 0.03	1225	1.43

Monosaccharide composition analysis further showed that SLPPs consisted of Man, GlcN, GlcA, Glc, and Gal, with a molar ratio of 1.002: 0.244: 9.121: 88.145: 1.488 (Fig. S1C, Table S2). Among these monosaccharides, Glc was the predominant residue, accounting for the vast majority of the total monosaccharide composition, indicating that SLPPs is a glucose-rich polysaccharide. This result is consistent with previous reports that polysaccharides from the *Sparassis* genus are mainly glucan-type macromolecules. Notably, the relatively high proportion of GlcA agrees well with the measured uronic acid content, further supporting that SLPPs is an acidic heteropolysaccharide rather than a neutral homopolysaccharide. The minor amounts of Man, Gal, and GlcN suggest that, in addition to the glucose-rich main framework, small amounts of other monosaccharide residues are also incorporated into the polysaccharide structure.

The glucose-dominant composition of SLPPs may be structurally relevant to its gel-forming behavior, because glucan-rich polysaccharides are prone to intermolecular association and chain entanglement, especially when combined with high molecular weight. Meanwhile, the presence of GlcA provides carboxyl groups that can enhance hydration capacity and may contribute to acid-triggered gelation by reducing electrostatic repulsion after protonation in the citric-acid system. These acidic groups may also participate in metal-ion chelation and hydrogen-bonding interactions, which could partly explain the antioxidant potential and water-management performance of the SLPPs hydrogel observed in this study. Therefore, the combined features of a glucose-rich backbone, appreciable uronic acid content, and ultra-high molecular weight likely constitute an important structural basis for the favorable gelation and functional properties of SLPPs.

### 3.3 Gelation and thermoreversible behavior of SLPPs hydrogel

The gelation capability of SLPPs was successfully induced by *CA.* A stable, transparent hydrogel was formed (Fig. 1B). Under the selected formulation condition, the apparent pH of the hydrogel dispersion was 2.76 ± 0.09, confirming that gel formation occurred under strongly acidic conditions. This represents the first report of citric-acid-induced gelation within polysaccharides derived from *S. latifolia* pseudosclerotia. The successful formation of a transparent hydrogel induced by CA underscores the potential of SLPPs as a novel gelling agent. The transparency suggests a homogeneous and fine-stranded gel network, which is often associated with polysaccharides that form junction zones *via* non-covalent interactions, such as hydrogen bonding and hydrophobic associations (Li, Liu, et al., 2023).

The obtained SLPPs hydrogel exhibited notable thermoreversibility. Upon heating, the hydrogel progressively lost its macroscopic integrity, becoming semi-fluid at approximately 80 °C and completely fluid at 90 °C (Fig. 1C). Subsequent cooling at 4 °C restored a self-supporting hydrogel. This visually reversible gel–sol–gel transformation is consistent with the reversible disruption and reformation of physical junction zones during heating and cooling. This heating-cooling cycle could be repeated multiple times, demonstrating reproducible macroscopic thermoreversibility under the tested heating–cooling conditions. The thermoreversible behavior observed—melting upon heating and re-gelation upon cooling—is a characteristic feature of physical gels, where the network is stabilized by reversible physical crosslinks. The melting of the gel at elevated temperatures (80–90 °C) can be attributed to the disruption of these physical junctions. The mechanism behind this behavior likely involves the complex structure of SLPPs, particularly the *β*-glucan backbone with (1 → 3)- and (1 → 6)-linkages. Similar thermoreversible gels have been reported for *S. crispa β*-glucan, where the gelation is driven by the formation and dissociation of helical aggregates and chain associations in response to temperature changes (Furuta et al., 2023). This thermoreversible property is highly beneficial for practical applications. In the food industry, it enables easy processing, filling, and sterilization at high temperatures, after which the material sets into a gel upon cooling. This characteristic is ideal for products like desserts and sauces and for encapsulating heat-sensitive bioactives (Kierulf et al., 2022). Furthermore, the ability to endure multiple gel-sol-gel cycles also points to the material's good stability and reprocessability. The present results establish the gel-forming capability of SLPPs under the selected formulation condition. Because acid concentration, polymer concentration, thermal treatment, and cooling history can independently influence polysaccharide association, systematic mapping of the gelation window will be required before formulation optimization or industrial process parameters can be defined.

### 3.4 WHC and SR of SLPPs hydrogel

**Fig. 3:**
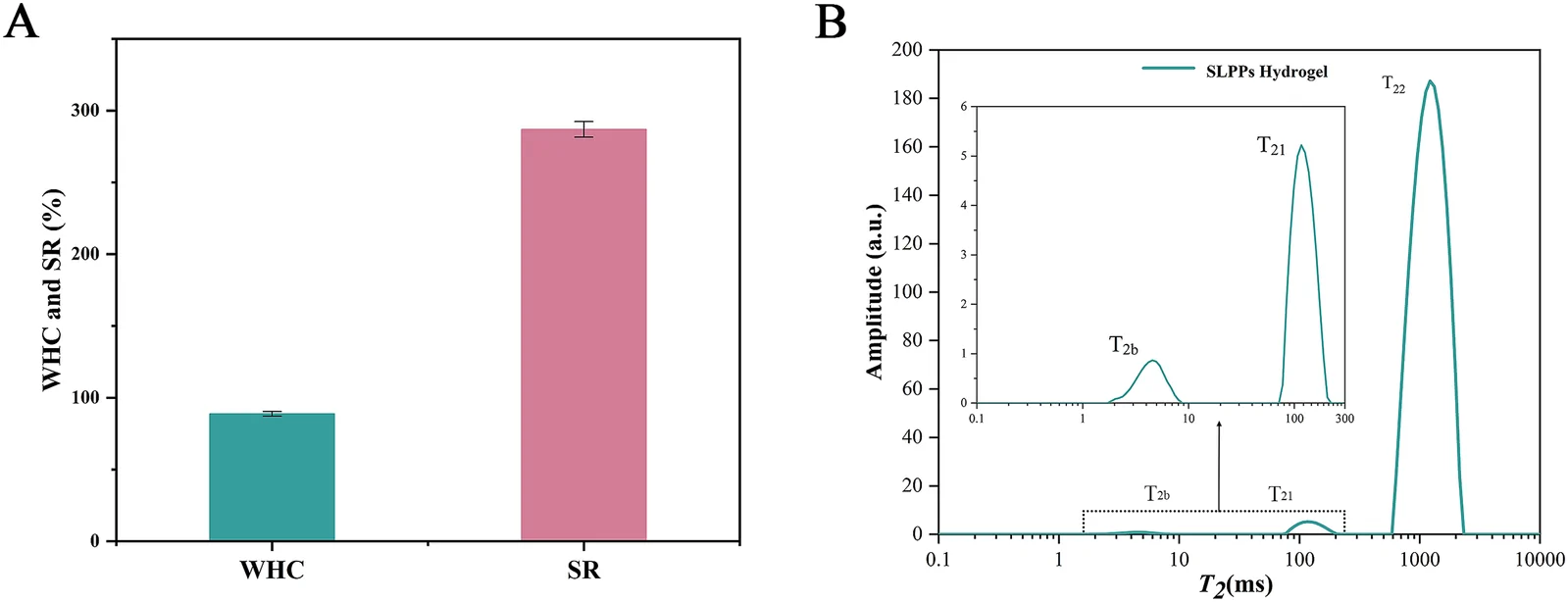
Water-holding capacity (WHC), swelling ratio (SR), and LF-NMR analysis of SLPPs hydrogel. (A) WHC and SR. (B) Water distribution within the SLPPs hydrogel.

The WHC and SR are critical parameters for evaluating the practical potential of hydrogels. The SLPPs hydrogel exhibited an exceptionally high WHC of 89% (Fig. 3A). This outstanding value indicates a robust ability to retain water under mechanical stress, attesting to the strength and stability of its three-dimensional network (Saha & Bhattacharya, 2010). A high WHC suggests that the gel matrix can effectively immobilize a large amount of water through capillary forces and strong interactions with the hydrophilic groups of the polysaccharide chains, mitigating syneresis under mechanical stress. This property is highly desirable in food applications, for instance, in preventing juice leakage in gel-based desserts, improving the juiciness of structured foods, and enhancing the shelf-life and stability of products (Ma et al., 2023). The ability to retain water so effectively can be attributed to the dense and homogeneous network structure formed by the high molecular weight *β*-glucans through acid-induced association, which creates numerous small pores that can trap water efficiently (Wanniarachchi et al., 2025).

The SLPPs hydrogel reached its swelling equilibrium rapidly, within 15 min, achieving a remarkable maximum SR of 280% (Fig. 3A), indicating the hydrophilic nature and the porous microstructure of the gel network. The relatively high equilibrium swelling ratio further confirms the network's capacity to absorb and hold a significant amount of water, which is a crucial characteristic for applications such as wound dressings that require exudate absorption or as a delivery system for water-soluble bioactive compounds (Gounden & Singh, 2024). The combination of high WHC and rapid, substantial swelling suggests that the SLPPs hydrogel possesses a cohesive yet flexible network. The network is strong enough to resist collapse and water expulsion under mechanical force (high WHC), while also being sufficiently hydrophilic and porous to allow for rapid water penetration and substantial volume expansion (high SR) (Feng & Wang, 2023). This balance between mechanical stability and hydrophilicity positions the SLPPs hydrogel as a highly functional material with significant potential across food, cosmetic, and pharmaceutical industries.

### 3.5 LF-NMR analysis of SLPPs hydrogel

The state and distribution of water within the SLPPs hydrogel were investigated using LF-NMR, with the spin-spin relaxation time (*T*_2_) distribution presented in Fig. 3B. The *T*_2_ relaxation spectrum displayed three distinct peaks, labeled *T*_2b_, *T*_21_, and *T*_22_, which correspond to water populations in different physical states within the gel matrix. *T*_2b_ corresponds to tightly bound water strongly associated with polar groups on the polysaccharide chains through hydrogen bonding and specific adsorption (Gun'ko et al., 2017). Its short relaxation times indicate severely restricted mobility at polymer–water interfaces. *T*_21_ represents weakly bound or capillary water located in small pores and inter-fibrillar spaces, where translational motion is partially hindered by confinement and surface relaxation. The prominent *T*_22_ component is assigned to bulk-like or free water residing in larger aqueous domains that remain held within the continuous hydrogel matrix (Cortez-Trejo et al., 2022). The relatively high amplitude and narrow width of *T*_22_ imply that most water experiences similar, moderately constrained environments, consistent with a homogeneous network with mesoscale pores. This hierarchical distribution is characteristic of physically cross-linked hydrogels and supports the view that SLPPs form a hydrated three-dimensional network capable of retaining substantial amounts of water while limiting macroscopic migration (Li et al., 2016). The existence of *T*_2b_ and *T*_21_, albeit minor, is crucial for WHC and syneresis resistance, because these fractions act as “anchors” that reduce drainage under stress or storage. Meanwhile, the predominance of *T*_22_ explains the high overall water content and contributes to desirable softness and juiciness in hydrocolloid applications (Li et al., 2022). Taken together, LF-NMR reveals that SLPPs generate a relatively uniform yet sufficiently interactive matrix that balances water immobilization with bulk hydration—an advantageous combination for designing food hydrogels with high WHC and low phase separation.

### 3.6 Ft-IR

**Fig. 4:**
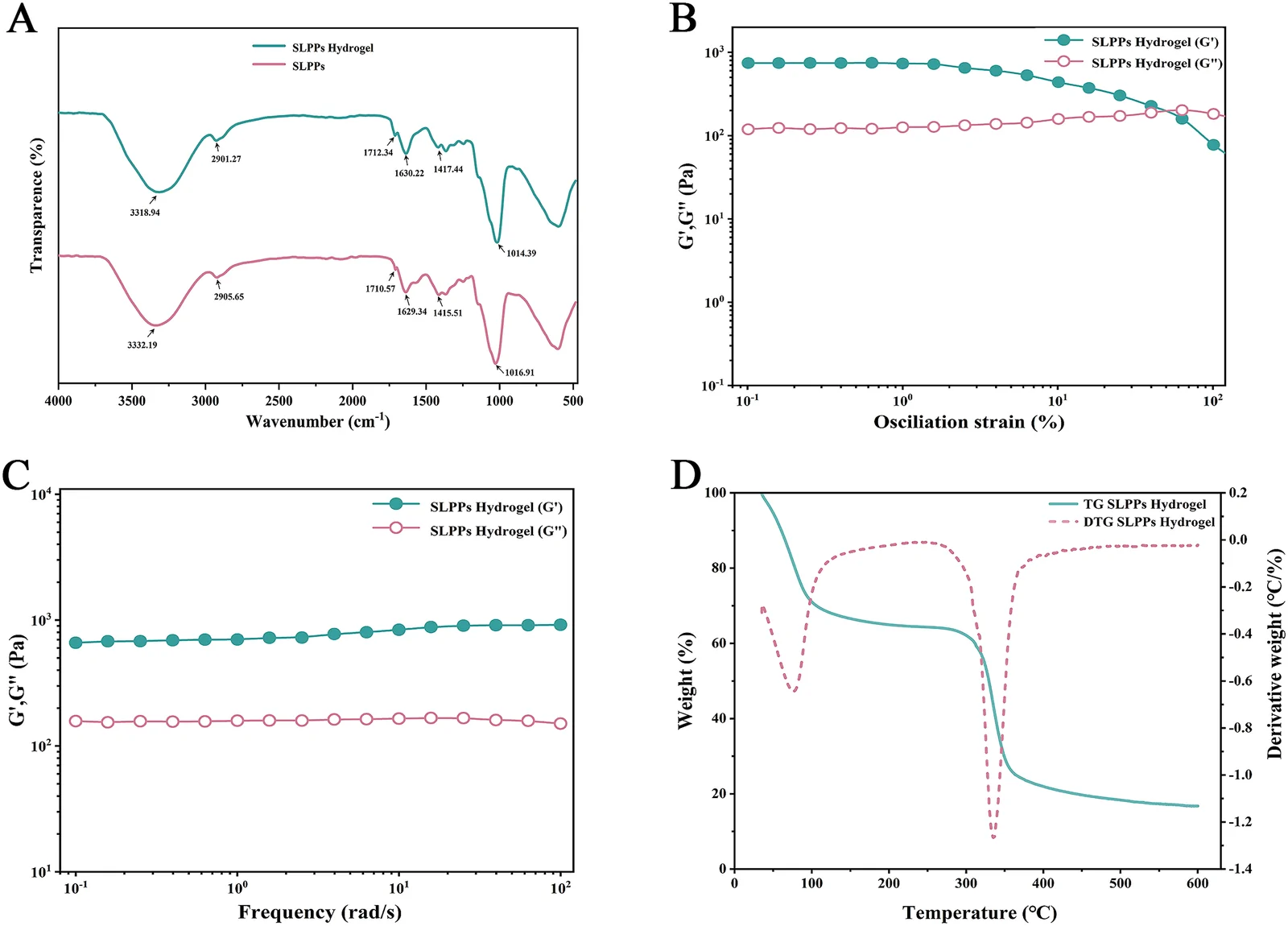
Characterization of the structural, rheological, and thermal properties of the SLPPs hydrogel (A) FT-IR spectra of SLPPs powder and the resulting SLPPs hydrogel. (B) Amplitude sweep tests showing the linear viscoelastic region (LVR). (C) Dynamic frequency sweep measurements of storage modulus (*G*′) and loss modulus (*G*″). (D) Thermogravimetric analysis.

FT-IR spectroscopy was used to characterize the functional groups of SLPPs and to evaluate changes in the molecular interaction environment associated with hydrogel formation (Fig. 4A). Both SLPPs and the corresponding hydrogel exhibited typical absorption features of polysaccharides, with broadly similar spectral profiles. The broad bands at 3318.94 and 3332.19 cm^−1^ were assigned to O—H stretching vibrations, reflecting the abundance of hydroxyl groups and the extensive hydrogen-bonding environment within the polysaccharide system. The absorptions at 2901.27 and 2905.65 cm^−1^ were attributed to C—H stretching vibrations of the carbohydrate skeleton. Bands at 1712.34 and 1710.57 cm^−1^ were associated with protonated and/or hydrogen-bonded carboxyl groups under the acidic conditions, whereas those at 1630.22 and 1629.34 cm^−1^ were consistent with carbonyl-related vibrations associated with uronic-acid-containing residues. In addition, the bands at 1417.44 and 1415.51 cm^−1^ were assigned to symmetric stretching vibrations of carboxylate groups, in agreement with the presence of uronic acid in SLPPs. Strong absorptions at 1014.39 and 1016.91 cm^−1^ were attributed to C–O–C and C—O stretching vibrations within the pyranose ring structure, further confirming the characteristic polysaccharide framework of SLPPs (Ren et al., 2022).

Notably, hydrogel formation did not generate new characteristic absorption bands, and the principal polysaccharide-associated bands remained at comparable positions before and after gelation. This indicates that the citric-acid-assisted gelation process did not involve the formation of readily detectable new covalent linkages. Instead, the broad O—H absorption together with the carboxyl-related bands suggests that rearrangement of hydrogen-bonding environments and interactions involving ionizable carboxyl groups accompanied network formation. The strongly acidic environment generated by citric acid may increase the protonation of carboxyl groups, thereby decreasing electrostatic repulsion between adjacent polysaccharide chains and facilitating closer intermolecular association. Meanwhile, the persistence of the characteristic polysaccharide backbone signals indicates that the primary chemical framework of SLPPs was largely retained during gel formation. Overall, the FT-IR characteristics are consistent with the formation of a predominantly physically associated network stabilized by reversible noncovalent interactions rather than covalent crosslinking, which is characteristic of many polysaccharide-based physical hydrogels (Guo et al., 2024).

### 3.7 Textural properties of SLPPs hydrogel

Textural characteristics, including hardness, chewiness, elasticity, cohesion, and gel strength, can systematically reflect the properties of polysaccharide-based hydrogel systems (Sarkar et al., 2021). As shown in Table 2 and Video 1, the SLPPs hydrogel is a soft yet elastic material with moderate hardness. Its comparatively high springiness indicates substantial elastic recovery after compression. Moreover, the relatively low chewiness, together with the high springiness and measurable cohesiveness, is consistent with a soft, water-rich network capable of recovering from moderate deformation. Such a mechanical response is characteristic of physically associated polysaccharide gels in which reversible junction zones and chain entanglement permit deformation without immediate structural collapse (Rumon et al., 2024). Coupled with the modest gel strength, these textural characteristics indicate a cohesive but compliant matrix, which may be advantageous for spreadable or spoonable foods and for encapsulation systems requiring stress dissipation (Russo Spena et al., 2024).

**Table 2:** Texture properties of SLPPs hydrogel.

Sample	Hardness (g)	Springiness (mm)	Chewiness (mJ)	Cohesiveness (%)	Gel strength (g)
SLPPs hydrogel	71.60 ± 1.90	2.77 ± 0.12	8.49 ± 0.10	0.64 ± 0.40	13.10 ± 0.25

### 3.8 Rheological properties

The viscoelastic properties of the SLPPs hydrogel—crucial for understanding its mechanical robustness and application potential—were characterized by oscillatory rheological measurements.

#### 3.8.1 Linear viscoelastic range

The LVR of the SLPPs hydrogel was determined by amplitude-sweep measurements (Fig. 4B). Both *G′* and *G″* remained relatively constant up to a strain of approximately 1%, indicating that the internal network was maintained within this deformation range. Above this critical strain, G′ decreased rapidly, indicating progressive disruption of the gel structure. A defined LVR is characteristic of structured biopolymer gels and provides an appropriate strain window for probing their intrinsic viscoelastic response without inducing substantial structural damage (Ata et al., 2025). Consequently, all subsequent frequency sweep measurements were conducted within this 1% strain limit to ensure the analysis of the undisturbed gel structure, a standard practice for accurately characterizing the intrinsic properties of soft solid materials.

#### 3.8.2 Dynamic rheology

The frequency-dependent viscoelastic behavior of the SLPPs hydrogel was evaluated within the LVR (Fig. 4C). Across the tested angular-frequency range of 0.1–100 rad·s^−1^, *G′* remained consistently higher than *G″*, with an approximately one-order-of-magnitude separation between the two moduli. This elastic-dominant response is characteristic of a structured, solid-like gel in which stress is stored more effectively than it is dissipated (Ata et al., 2025). Both moduli exhibited relatively weak frequency dependence: *G′* increased moderately with angular frequency, whereas *G″* remained comparatively stable. Such limited frequency dependence indicates that the network persisted across the investigated timescales and is consistent with a physically associated polysaccharide gel containing relatively long-lived junction zones (Müller et al., 2025). These rheological characteristics demonstrate the mechanical integrity of the matured SLPPs hydrogel under the selected formulation condition, although the present frequency-sweep measurements do not by themselves resolve the molecular origin of the junctions or their dependence on pH and temperature.

### 3.9 TGA

The SLPPs hydrogel exhibited three distinct weight-loss stages upon heating (Fig. 4D). The first stage (40–150 °C) was characterized by a rapid mass loss of ∼30–35%, accompanied by a shallow DTG minimum near 80 °C, and was attributed to the removal of free and crystalline water together with a small amount of volatile species physically trapped within the network (Zhao et al., 2025). The second weight-loss stage (150–400 °C) was characterized by the most pronounced mass loss, bringing the cumulative loss to ∼40–45%. The DTG exhibited a strong peak at ∼340 °C, indicating the onset of structural degradation of the polysaccharide matrix—namely, cleavage of glycosidic linkages, dehydration/condensation reactions, and partial decarboxylation of uronic units (Rong et al., 2021). The third weight-loss stage (400–600 °C) involved a slow additional loss (∼8–10%), yielding a final residue of ∼17% at 600 °C, consistent with carbonization/aromatization and the progressive decomposition of thermally stable fragments, leaving a carbonaceous/ash residue. Overall, the TGA/DTG data indicated that the SLPPs hydrogel maintained practical thermal integrity up to ∼300 °C; above this temperature, backbone degradation dominated. This behavior was consistent with that reported for other uronic-acid-rich polysaccharide networks. Understanding these thermal boundaries is essential for tailoring applications of the SLPPs hydrogel in food, cosmetic, and pharmaceutical products, where thermal processing is a critical consideration.

### 3.10 Microstructure of SLPPs hydrogel

**Fig. 5:**
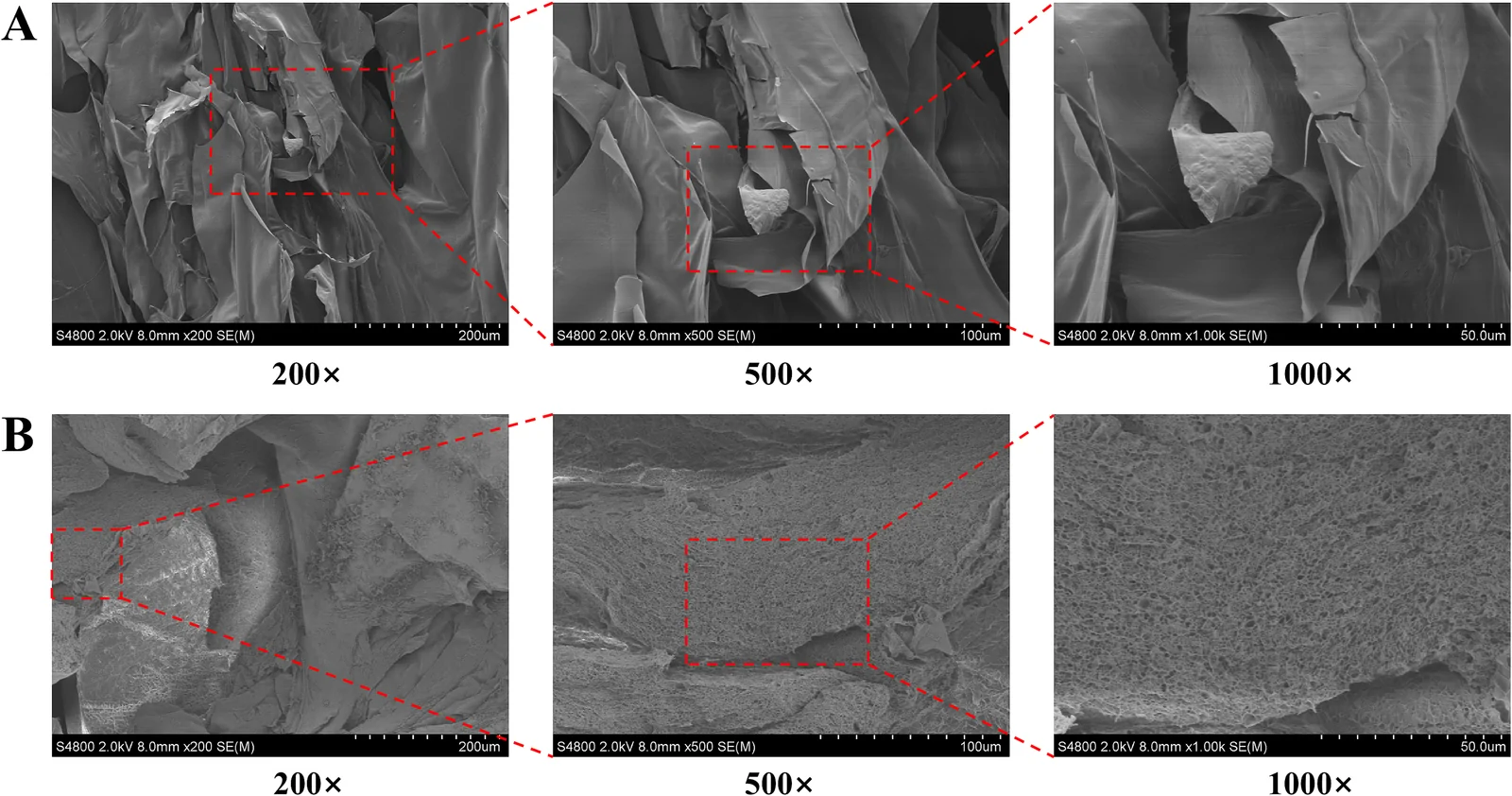
SEM micrographs of lyophilized SLPPs (A) and SLPPs hydrogel (B) at 200×, 500×, and 1000× magnifications. Dashed boxes and connecting lines indicate the regions examined at higher magnification. Representative folded lamellar structures in SLPPs and the interconnected porous network in the hydrogel are indicated in the corresponding micrographs.

The microstructural features of the SLPPs powder and the corresponding citric-acid-induced hydrogel were examined by SEM (Fig. 5). The SLPPs powder exhibited a relatively dense, smooth, and planar surface with no visibly distinct porosity, typical of polysaccharide powders prior to hydration and network formation (Chen et al., 2022). By contrast, the SLPPs hydrogel displayed a highly porous, three-dimensional network. The gel matrix featured numerous interconnected pores, a markedly rougher surface, and folded, sheet-like lamellae. This pronounced transformation—from a dense powder to an open, porous network—arises directly from gelation. The formation of this interconnected, porous structure is characteristic of physically cross-linked polysaccharide hydrogels and is crucial for water uptake and retention, consistent with the high WHC and SR previously observed for this hydrogel (Li et al., 2023b). The well-defined, continuous network confers the structural integrity and stability required for mechanical strength, effectively explaining the solid-like rheological behavior in which the *G*′ exceeds the *G*″. This coherent microstructure underscores the success of the citric-acid-induced gelation strategy and highlights the potential of the SLPPs hydrogel for applications such as controlled-release systems and as a scaffold in food matrices, where high surface area and interstitial volume are advantageous.

### 3.11 Proposed gelation mechanism of SLPPs hydrogel

**Fig. 6:**
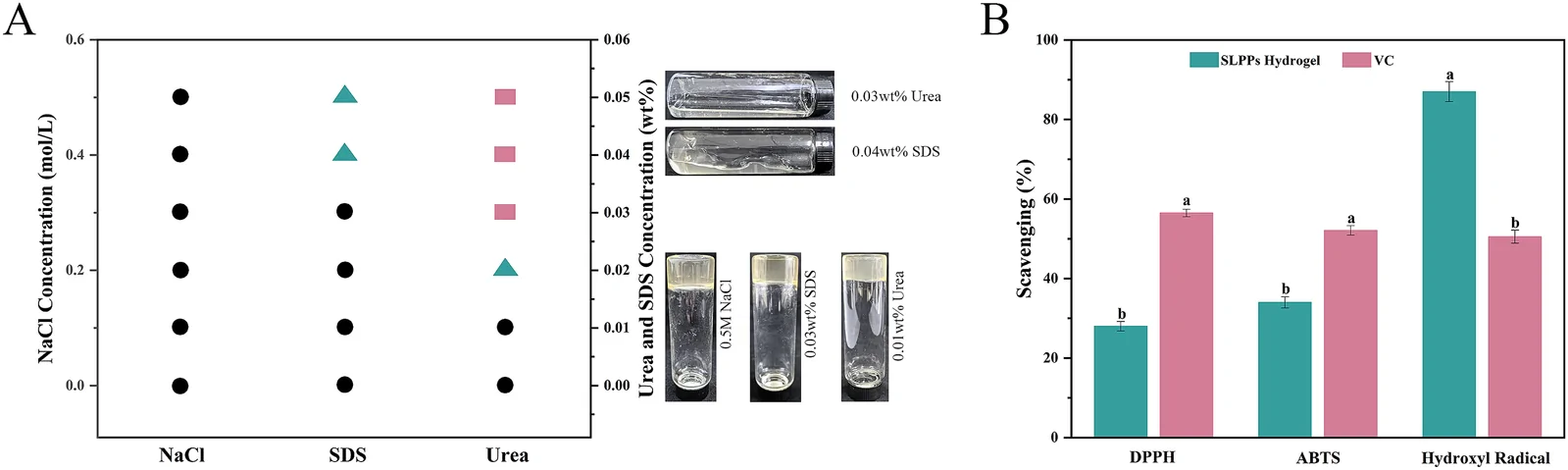
Salt/denaturant sensitivity and antioxidant activities of SLPPs hydrogel. (A) Effects of NaCl, SDS, and urea on SLPPs hydrogel integrity, (●) represents the gel state; () represents the semi-gel; () represents the liquid state. (B) Antioxidant activities.

Selective disruption experiments provided complementary phenomenological evidence regarding the noncovalent interactions contributing to the stability of the SLPPs hydrogel network. Increasing NaCl concentrations from 0 to 0.5 M caused no obvious change in gel integrity, whereas the addition of SDS or urea progressively weakened the hydrogel and ultimately converted it into a flowable system (Fig. 6A). Because SDS can disrupt hydrophobic association whereas urea interferes with hydrogen-bonding interactions, the differential sensitivity of the hydrogel to these reagents supports important contributions from hydrophobic association and hydrogen bonding to network stabilization. This interpretation is consistent with the FT-IR results, in which no new characteristic absorption bands appeared after gel formation, indicating the absence of detectable new covalent linkages. The reversible gel–sol–gel transition observed upon heating to 80–90 °C and subsequent cooling further supports a predominantly physically cross-linked network stabilized by reversible intermolecular interactions.

CA appears to play an important role in facilitating the initial association of SLPPs chains. The 10% (*w*/*v*) citric acid solution used for gel preparation corresponded to approximately 0.52 mol L^−1^, and the apparent pH of the hydrogel dispersion was 2.76 ± 0.09, indicating that gelation occurred under strongly acidic conditions. SLPPs contained 9.74% uronic acid, providing ionizable carboxyl groups within the polysaccharide chains. Under this acidic environment, increased protonation of these groups would be expected to reduce intermolecular electrostatic repulsion and allow closer chain–chain approach. At the same time, heating at 85 °C increases molecular mobility and disrupts pre-existing weak associations, allowing the high-molecular-weight SLPPs chains (*Mw* = 1225 kDa) to undergo conformational rearrangement. During subsequent cooling at 4 °C, reduced thermal motion favors the re-establishment of intermolecular hydrogen bonds and hydrophobic contacts, while extensive chain entanglement associated with the high molecular weight of SLPPs further contributes to the formation of a continuous three-dimensional network. Thus, citric acid is more appropriately considered a gelation-promoting agent that creates a favorable acidic environment for intermolecular association rather than a conventional covalent crosslinker.

**Fig. 7:**
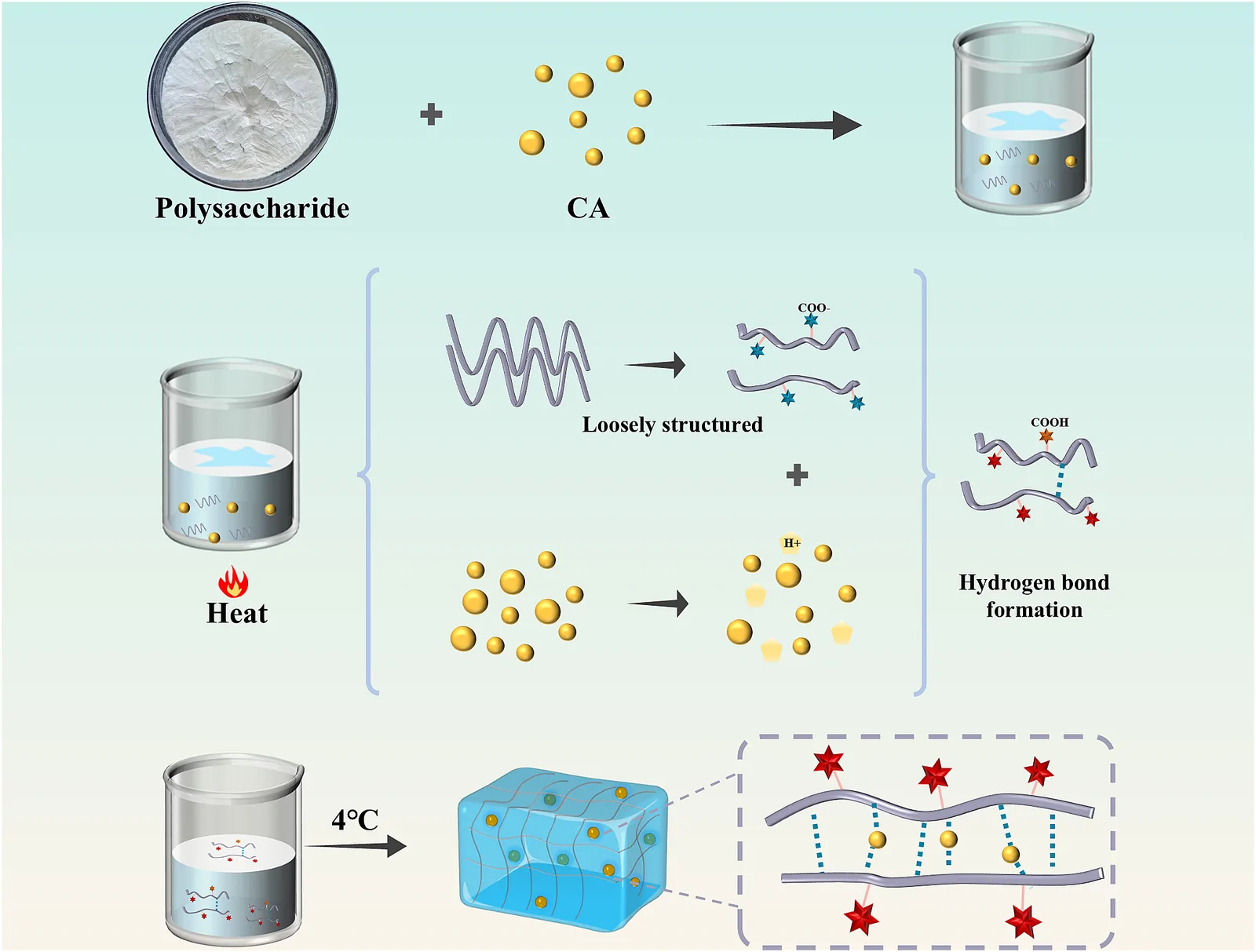
Proposed gelation mechanism during SLPPs hydrogel formation.

This interpretation is further supported by the macroscopic and physicochemical characteristics of the resulting hydrogel. The predominance of *G′* over *G″* across the tested frequency range demonstrates the formation of a stable, elastic network, while LF-NMR revealed tightly bound, weakly bound, and bulk-like water populations within the gel matrix. The high water-holding capacity and rapid swelling are therefore consistent with a hydrated network capable of simultaneously immobilizing water and permitting water exchange. SEM observation further showed that gelation transformed the relatively compact SLPPs morphology into an interconnected porous architecture, providing structural support for these water-management properties. Taken together, the chemical composition, interaction-disruption, spectroscopic, rheological, thermal, LF-NMR, and microstructural results support a gelation model in which citric-acid-assisted reduction of interchain repulsion facilitates molecular approach, followed by thermally induced chain rearrangement and the formation of reversible hydrogen-bonded and hydrophobically associated junctions together with high-molecular-weight chain entanglement during cooling (Fig. 7). Nevertheless, the present dataset does not quantitatively resolve the relative contribution of individual intermolecular forces. In particular, ζ-potential–pH relationships and pH- and temperature-resolved rheological evolution were not determined. Accordingly, the proposed mechanism is interpreted as a model consistent with the convergent experimental evidence rather than as a quantitatively resolved molecular mechanism.

### 3.12 Antioxidant activities *in vitro*

The *in vitro* antioxidant capacity of the washed SLPPs hydrogel was evaluated against DPPH, ABTS, and hydroxyl radicals (Fig. 6B). After gel formation, the hydrogel was thoroughly washed with deionized water to remove free and readily diffusible citric acid, resulting in an increase in the apparent pH of the hydrogel dispersion from 2.76 ± 0.09 before washing to 5.26 ± 0.06 after washing. Preliminary control experiments further showed that variation within pH 5.0–6.0 did not significantly alter the baseline absorbance of the DPPH or ABTS assay systems. These observations indicate that residual acidity was unlikely to substantially influence the DPPH and ABTS responses observed for the washed hydrogel. The SLPPs hydrogel exhibited a DPPH radical scavenging response of 28.0%. The relatively moderate response may partly reflect restricted diffusion of DPPH into the hydrated polysaccharide network and limited accessibility of reactive groups immobilized within the gel matrix. Unlike freely dissolved low-molecular-weight antioxidants, functional groups located within a cross-linked macromolecular network are subject to steric and mass-transfer constraints, which can reduce the apparent reaction rate within a fixed incubation period (Dziadek et al., 2022).

In the ABTS assay, the SLPPs hydrogel exhibited a scavenging activity of 34.0%, while VC showed a higher activity of 52.1%. The comparatively greater response toward ABTS than DPPH may reflect differences in radical accessibility, polarity, and reaction mechanism, together with the availability of hydroxyl- and carboxyl-containing groups within the polysaccharide matrix (Shahidi & Samarasinghe, 2025). As with the DPPH assay, the physical state of the hydrogel is likely to influence the apparent response through diffusion and accessibility effects.

A markedly stronger response was observed in the hydroxyl-radical assay, where the washed hydrogel produced 87.0% inhibition under the tested conditions. Hydroxyl-radical generation in this assay depends on Fe^2+^-mediated Fenton chemistry; therefore, the response may arise from a combination of direct radical scavenging and interactions between metal ions and functional groups within the polysaccharide matrix (Abe et al., 2022). This potent activity may be linked to the specific structural features of SLPPs, such as its high molecular weight and the spatial arrangement of functional groups within the gel matrix. Taken together, SLPPs hydrogel possesses substantial and multifaceted *in vitro* antioxidant properties. Its particularly strong efficacy against the highly damaging hydroxyl radical, coupled with moderate activity toward DPPH and ABTS radicals, highlights its potential as a functional biomaterial. These findings suggest that SLPPs hydrogel is a promising candidate for applications such as antioxidant-active food packaging, cosmetic formulations aimed at protecting skin from oxidative damage, and biomedical settings where mitigation of oxidative stress is crucial. However, because the hydrogel and VC differ fundamentally in physical state and mass-transfer behavior, VC should be regarded as an assay-performance reference rather than a matrix-equivalent benchmark of intrinsic antioxidant potency.

### 3.13 Antioxidant activities *in vivo*

To further substantiate the antioxidant potential of the SLPPs hydrogel at the organism level, serum and tissue redox-related biomarkers were quantified after 30 days of oral administration, including the enzymatic antioxidants SOD and CAT and the lipid peroxidation product MDA. Throughout the 30-day intervention, no mortality or overt treatment-related abnormalities in the general condition or behavior of the animals were observed. The administered hydrogel doses (30, 120, and 300 mg·kg^−1^ body weight) were selected to span a broad exposure range around doses previously reported for *S. latifolia* polysaccharides. Oral doses of 100–400 mg·kg^−1^ have been used in previous mouse studies investigating antioxidant-related and immunomodulatory effects of *S. latifolia* polysaccharides (He et al., 2023). The present design therefore allowed the response to be examined from a comparatively low exposure to a dose within the previously studied upper range. Overall, the SLPPs hydrogel administration produced dose-associated changes in serum and tissue redox-related biomarkers, characterized by increased SOD and CAT activities and decreased MDA levels. Importantly, the inclusion of the VC group as a positive control provided a useful benchmark for evaluating the efficacy of the hydrogel treatment (Table 3, Table 4, Table 5).

**Table 3:** Serum antioxidant enzyme activities (SOD and CAT) and MDA in mice.

Treatments	SOD (**U**·**mL**^−1^)	CAT (**U**·**mL**^−1^)	MDA (nmol·**mL**^−1^)
NC	270.3 ± 16.8 ^c^	108.6 ± 10.2 ^d^	9.2 ± 1.8 ^a^
VC	415.6 ± 10.4 ^a^	238.5 ± 12.5 a	5.0 ± 0.4 ^c^
HL	290.5 ± 15.2 ^c^	132.5 ± 14.8 ^c^	7.5 ± 0.8 ^b^
HM	340.8 ± 22.3 ^b^	192.3 ± 19.2 ^b^	5.9 ± 1.1 ^c^
HH	405.6 ± 20.5 ^a^	232.5 ± 17.6 ^a^	5.3 ± 0.6 ^c^

Values are mean ± SD (*n* = 12). Normality and homogeneity of variance were confirmed before one-way ANOVA followed by Tukey's HSD test. Different lowercase superscript letters within a column indicate significant differences (*P* < 0.05).

**Table 4:** The liver's SOD and CAT activities and MDA level in mice.

Treatments	SOD (U·mgprot^−1^)	CAT (U·mgprot^−1^)	MDA (nmol·mgprot^−1^)
NC	102.4 ± 11.2 ^e^	226.4 ± 15.5 ^e^	8.3 ± 0.8 ^a^
VC	205.6 ± 12.6 ^a^	375.5 ± 13.3 ^a^	4.3 ± 0.3 ^d^
HL	125.3 ± 12.6 ^d^	252.6 ± 12.9 ^d^	7.2 ± 0.5 ^b^
HM	140.6 ± 15.3 ^c^	301.1 ± 19.8 ^c^	6.5 ± 0.4 ^b^
HH	186.6 ± 10.8 ^b^	352.8 ± 18.9 ^b^	5.1 ± 0.9 ^c^

Values are mean ± SD (*n* = 12). Normality and homogeneity of variance were confirmed before one-way ANOVA followed by Tukey's HSD test. Different lowercase superscript letters within a column indicate significant differences (*P* < 0.05).

**Table 5:** Splenic SOD and CAT activities and MDA level in mice.

Treatments	SOD (U·mgprot^−1^)	CAT (U·mgprot^−1^)	MDA (nmol·mgprot^−1^)
NC	155.3 ± 8.2 ^d^	286.2 ± 12.1 ^d^	8.6 ± 0.6 ^a^
VC	252.3 ± 12.5 ^a^	354.4 ± 12.1 ^a^	4.4 ± 0.3 ^d^
HL	176.8 ± 10.4 ^c^	303.1 ± 10.4 ^c^	7.7 ± 0.5 ^b^
HM	200.3 ± 9.6 ^b^	331.5 ± 12.8 ^b^	6.1 ± 1.2 ^c^
HH	246.6 ± 11.3 ^a^	346.8 ± 11.9 ^a^	4.7 ± 0.6 ^d^

Values are mean ± SD (*n* = 12). Normality and homogeneity of variance were confirmed before one-way ANOVA followed by Tukey's HSD test. Different lowercase superscript letters within a column indicate significant differences (*P* < 0.05).

The systemic antioxidant status is effectively reflected by serum enzyme levels, which serve as the primary defense against circulating reactive oxygen species (ROS) (Marrocco et al., 2017). Compared with the NC group, SLPPs hydrogel administration progressively enhanced serum SOD and CAT activities while lowering MDA levels. In the HH group, serum SOD and CAT reached 405.6 and 232.5 U·mL^−1^, respectively, whereas serum MDA decreased to 5.3 nmol·mL^−1^. Notably, these values were very close to those of the VC group (415.6 U·mL^−1^ for SOD, 238.5 U·mL^−1^ for CAT, and 5.0 nmol·mL^−1^ for MDA), and no significant differences were observed between HH and VC within the same column. This result indicates that high-dose SLPPs hydrogel was able to produced serum redox-related biomarker values comparable with those of the VC reference group. The HM group also showed marked improvement relative to NC, whereas the HL group produced only a moderate effect, further supporting a dose-dependent response (Zheng et al., 2023).

The liver, as the major metabolic and detoxification organ, is highly vulnerable to oxidative stress. In the present study, all hydrogel-treated groups showed improvement in hepatic antioxidant status compared with NC, as evidenced by increased SOD and CAT activities and decreased MDA levels. In the HH group, hepatic SOD and CAT activities increased to 186.6 and 352.8 U·mgprot^−1^, respectively, while MDA decreased to 5.1 nmol·mgprot^−1^. Although these values were significantly improved relative to NC, they remained below those of the VC group (205.6 U·mgprot^−1^ for SOD, 375.5 U·mgprot^−1^ for CAT, and 4.3 nmol·mgprot^−1^ for MDA). This suggests that SLPPs hydrogel produced dose-related changes in hepatic antioxidant-related biomarkers, but its efficacy in liver tissue did not fully reach that of the VC reference group under the present conditions. Therefore, the hepatic response appears to be dose-dependent but somewhat less sensitive than the serum response.

The spleen, a major peripheral immune organ, also showed a pronounced improvement in redox status following hydrogel administration. In the HH group, splenic SOD and CAT activities increased to 246.6 and 346.8 U·mgprot^−1^, respectively, and MDA decreased to 4.7 nmol·mgprot^−1^. These values were highly comparable to those of the VC group (252.3 U·mgprot^−1^ for SOD, 354.4 U·mgprot^−1^ for CAT, and 4.4 nmol·mgprot^−1^ for MDA), with the same significance grouping observed for all three indicators. This finding indicates that high-dose SLPPs hydrogel yielded values statistically comparable with those observed in the VC reference group under the present experimental conditions. Given the known immunomodulatory activity of *Sparassis β*-glucans, the strong splenic response may be related not only to direct antioxidation but also to the preservation of immune-cell redox homeostasis (Ohno et al., 2000). The observed dose-dependent responses across serum, liver, and spleen confirm that the SLPPs hydrogel possesses dose-associated modulation of antioxidant-related biomarkers *in vivo*.

The observed *in vivo* responses of SLPPs hydrogel can be attributed to its unique structural features and the delivery advantages of the hydrogel matrix. SLPPs are high-molecular-weight polysaccharides (1225 kDa) containing significant uronic acid (9.74%), which provides abundant hydroxyl and carboxyl groups for direct radical quenching and metal ion chelation. Beyond direct scavenging, mushroom polysaccharides are recognized for their ability to activate the nuclear factor erythroid 2-related factor 2 (Nrf2) signaling pathway. Upon oral administration, SLPPs may interact with pattern recognition receptors (*e.g.*, Dectin-1 or TLR4) on intestinal immune cells, triggering intracellular signaling that facilitates Nrf2 nuclear translocation and subsequently upregulates the transcription of downstream antioxidant genes such as SOD and CAT (Yu et al., 2021). Moreover, the citric-acid-induced hydrogel structure may play a protective role, potentially enhancing the stability of the polysaccharides during gastric transit and facilitating a more sustained interaction with gut receptors. This structural valorization not only converts a mushroom by-product into a functional hydrocolloid but also ensures that its biological potency is accompanied by measurable changes in antioxidant-related biomarkers *in vivo*.

Overall, the dose-associated changes observed in serum, liver, and spleen indicate that repeated administration of the washed SLPPs hydrogel modulated antioxidant-related biomarkers under the present experimental conditions. Although no overt adverse effects were observed during the 30-day intervention, the present study was not designed as a formal toxicological assessment. Therefore, dedicated cytotoxicity, histopathological, and systemic safety studies will be required before its suitability for long-term oral or pharmaceutical applications can be established.

## 4 Conclusion

In summary, this study demonstrates that polysaccharides recovered from *S. latifolia* pseudosclerotia can form transparent and thermoreversible hydrogels under a defined citric-acid-assisted condition. The resulting hydrogel exhibited predominantly elastic rheological behavior, high water-retention capacity, rapid swelling, and an interconnected porous microstructure. FT-IR spectroscopy, selective disruption experiments, thermal reversibility, and rheological observations collectively support a predominantly physically associated network involving reversible noncovalent interactions and high-molecular-weight chain entanglement. The strongly acidic environment of the formulation may facilitate closer intermolecular association; however, because effective ionic strength, residual citric acid, and pH-matched alternative-acid controls were not independently evaluated, the respective contributions of proton activity and citrate-specific effects remain unresolved. Crucially, the biological potency of the SLPPs was preserved and effectively translated into an *in vivo* setting, where oral administration significantly bolstered the antioxidant defense systems in the serum, liver, and spleen of mice. This research bridges the gap between agricultural waste upcycling and the design of bioactive food hydrocolloids. The SLPPs hydrogel serves as a promising material platform for the food, cosmetic, and pharmaceutical industries, offering a sustainable alternative for nutrient encapsulation and textural modification. Future work should focus on the interaction between the hydrogel matrix and the gut microbiota to further elucidate the molecular mechanisms underlying its health-promoting effects, thereby enhancing the overall value chain of the *Sparassis* mushroom industry.

The following are the supplementary data related to this article.

Supplementary video S1

Supplementary material

## CRediT authorship contribution statement

**Zhiheng Qiu:** Writing – original draft, Funding acquisition, Data curation. **Bei Jiang:** Writing – original draft, Methodology. **Di Zhou:** Validation. **Jiayang Hou:** Formal analysis. **Bo Bao:** Methodology. **Yongshun An:** Software. **Lu Ma:** Methodology. **Hao Liu:** Investigation. **Xiaoyan Zhang:** Software. **Jiazhi Zhao:** Resources. **Xia Ren:** Resources. **Tianlai Li:** Supervision. **Lili Shu:** Writing – review & editing, Validation, Resources, Methodology, Funding acquisition, Conceptualization.

## Ethics declaration

### Animal subject

This study was conducted in accordance with the ARRIVE (Animal Research: Reporting of *In Vivo* Experiments) guidelines. This study was approved by the Shenyang Agricultural University. (Approval No. SNLL26022701)

## Declaration of competing interest

The authors declare that they have no known competing financial interests or personal relationships that could have appeared to influence the work reported in this paper.

## Data Availability

Data will be made available on request.
