# Supplementary material for: Citric-acid-assisted upcycling of Sparassis latifolia pseudosclerotia into thermoreversible polysaccharide hydrogels: Structure–property relationships and antioxidant activity in vitro and in vivo
Source: Food Chem X. 2026 Sep 11;39:104447. doi: 10.1016/j.fochx.2026.104447 (PMC13594612; doi:10.1016/j.fochx.2026.104447)
Supplement: Supplementary file 1 [file mmc3.docx]

Table S1. Effect of pH 5.0–6.0 on DPPH and ABTS radical-scavenging activities.

| pH | DPPH radical scavenging rate (%) | ABTS radical scavenging rate (%) |
| --- | --- | --- |
| 5.00 | 27.69 ± 0.86 ^a^ | 34.19 ± 0.95 ^a^ |
| 5.25 | 28.00 ± 0.71 ^a^ | 33.27 ± 0.98 ^a^ |
| 5.50 | 27.30 ± 0.73 ^a^ | 34.73 ± 0.80 ^a^ |
| 5.75 | 28.33 ± 0.75 ^a^ | 34.48 ± 2.10 ^a^ |
| 6.00 | 28.29 ± 0.60 ^a^ | 34.12 ± 1.61 ^a^ |

Note: Values are expressed as mean ± SD (n = 3). Different lowercase letters within the same column indicate significant differences among pH treatments (*P* < 0.05); values sharing the same letter are not significantly different.

Table S2. Monosaccharide composition of SLPPs (molar ratio, %).

| Sample | Man | GlcN | GlcA | Glc | Gal |
| --- | --- | --- | --- | --- | --- |
| SLPPs | 1.002 | 0.244 | 9.121 | 88.145 | 1.488 |
